# Current and future developments in managed care in the United States and implications for Europe

**DOI:** 10.1186/1478-4505-3-4

**Published:** 2005-03-17

**Authors:** Ronald Lagoe, Deborah L Aspling, Gert P Westert

**Affiliations:** 1 Hospital Executive Council Syracuse, NewYork, USA; 2 Lodi Memorial Hospital Lodi, California, USA; 3 National Institute of Public Health and the Environment Bilthoven, Netherlands

## Abstract

The paper reviews and evaluates current and future approaches to cost containment in the United States. Managed care was once seen as an effective approach to supporting health care quality while containing         costs in the USA. In recent years payors started to look in other directions, since prospects for limiting expenses faded. Nowadays consumer driven health plans seem to be on the rise. The reasons for the decline of managed care, the growing popularity of the consumer driven health plans and the implications for Europe are discussed.

## Introduction

On an international basis, the development of health care policy is increasingly being influenced by cost considerations. Advances in health science and the delivery of care continue to expand the capabilities of treatments. The ability of nations and communities to pay for this care from available resources is a major subject of debate.

One focus of this debate has been research comparing health care utilization in Europe and the United States. Research has frequently demonstrated that, while Europe has greater capacity and higher utilization of services than the United States, Americans are paying more for these services. This discussion has intensified as nations on both sides of the Atlantic struggle to provide health and other programs while maintaining economic stability [1,2].

Frustrating as they may be for health care providers and payors, this situation presents important opportunities for research concerning health care policy. In particular, the experience of the United States during recent years, contains significant economic challenges with direct implications for health care financing and delivery. After eliminating federal budget deficits during the 1990s, America has generated a new round of shortfalls (exceeding $400 billion) annually through a combination of international military involvements and an economic downturn. This situation may impact Medicare, the major source of health care funding for the elderly and the largest single payor for these services [3,4].

Resource limitations are also challenging other major health care payors in the United States. Private insurance has carried the burden of cost shifting from public payors and has also suffered from the impact of the economic recession [4]. The Medicaid program, which funds health services for the indigent and elderly, has been challenged by budget difficulties of many state governments [5,6].

The pressures that are being exerted by these developments on the health care system of the United States promise to create opportunities for policy making concerning cost containment during the immediate future. The Bush Administration is already rediscovering Medicare managed care [7]. This type of activity could spill over to into other areas of the economy as the administration struggles to reconcile the costs of international commitments and domestic programs with its recent tax reductions.

Beyond these circumstances, current and future approaches to cost containment in the United States need to be viewed in a broader context. Managed care for a variety of payors was once seen as an effective approach to supporting health care quality while containing costs. During the past decade, the attractiveness of this approach to many employers has faded and prospects for limiting health care expenses have become confused [8]. These developments caused payors in America to look in other directions for approaches to containing health care expenses.

In this context, the recent experience of the United States with respect to health care and its economic impact may have valuable implications for that nation and for the rest of the world. In order to understand these implications, the development of managed care and other approaches to health care policy must be reviewed and evaluated. To provide a broad view of these changes, this information is presented as a review of existing sources and data rather than the development of completely new information.

## Development of Managed Care in the United States

Historically, managed care evolved in the United States to influence the use of medical care by improving outcomes and efficiency. The past three decades of experience with this approach suggests that at least some of these objectives have been realized.

During the second half of the twentieth century, managed care developed in the United States as a mechanism for constraining the growth of health care costs by controlling the delivery system. This approach originated in the western United States in the form of staff model plans such as Kaiser Permanente which employed physicians and other caregivers directly. In the private health insurance industry, managed care plans controlled costs and the delivery of care by restricting hospital utilization, such as admissions and lengths of stay, by limiting access to specialists, and by encouraging healthful behaviors among subscribers [9].

The popularity of managed care in the private sector of the American economy encouraged its adoption by public payors. The size of the Medicare program and the growth of health care expenses for the elderly stimulated the federal government to offer Medicare managed care options during the 1970s and 80s [10]. State governments, burdened with health care expenses for the indigent and elderly, and lacking the ability to run operating deficits, became even more interested in this approach [11].

During the 1980s and 90s, managed care expanded rapidly in the United States. In private health insurance, a major shift occurred from traditional indemnity insurance to managed care plans in many markets. This development was stimulated by an increase in the numbers of businesses offering managed care as an option to employees. The proportion of employees in large firms (those with more than 200 employees) enrolled in managed care plans grew from 5 percent in 1984 to 50 percent in 1993 [12]. As the use of managed care spread, interest in traditional indemnity plans declined. By 1998, only 14 percent of employees in large firms were enrolled in indemnity insurance plans [13].

During the 1980s and 90s, increased enrollment in private sector managed care in the United States was spurred by the ability of this approach to contain health care costs. The data in Table 1 demonstrate that, by 1997, average monthly insurance premiums for these plans were lower than for private health insurance. The data also indicate that the differences between premiums were greatest in areas where managed care penetration was lowest ($52 lower than non managed care plans) and in areas where managed care penetration was highest ($35 lower). This information suggests that managed care was successful in controlling costs in a variety of settings.

**Table 1:** Average monthly insurance premiums by type of insurance plan, 1997.

	Average Family Premiums ($)		
	All Plans	Managed Care	Non Managed Care
HMO Penetration			
Less than 25 Percent	439	401	453
25 – 35 Percent	417	408	423
35 – 45 Percent	400	400	401
45 Percent or More	394	380	415

Source: 1997 Robert Wood Johnson Foundation Employer Health Insurance Survey.

The expansion of managed care in the private sector of the United States was paralleled by increased adoption of this approach by public payors. Historically, the level of financial risk generated by the elderly and indigent, the major populations covered by Medicare and Medicaid, had limited government interest in privatizing these plans and the participation level of insurance companies in this area.

The failure of the Clinton administration to enact national health care reform did not impede the development of Medicare managed care. This approach expanded rapidly during the 1990's, fueled in part by the introduction of flexible plans. Enrollment in Medicare managed care, which had remained close to 1,000,000 between 1985 and 1991, increased to more than 6,000,000 by 1999 [14].

In the same period, State governments turned to managed care as a means of constraining health care costs. With fewer resources than the federal government, States had more incentives to use this approach. Between 1997 and 2001, enrollment in full risk Medicaid managed care plans increased by 40.6 percent [15].

The development of managed care in the United States was stimulated by interest in improving both outcomes and efficiency of health care. During the 1980s and 90s, the proliferation of this approach was related to pressure for efficiency and cost containment. In this role, managed care performed well throughout most of the two decades. As the use of this approach by all payors increased, the annual per cent change in per capita health spending in the nation declined from 5.0 – 6.9 between 1991 and 1993 to 2.0 – 2.2 between 1994 and 1996. Among private health insurance plans, where managed care penetration was highest, annual per capita changes in premiums declined from 12.5 percent in 1988 to 4.8 percent in 1997 [16].

Available evidence indicates that managed care was able to reduce health care expenses in the United States through constraints on utilization of service. From its beginnings, traditional managed care controlled utilization of hospital care, a major source of health care costs, through physician gate keeping and pre authorization mechanisms. In the western part of the nation, where use of this approach was highest, it supported hospital admission rates and length of stay that were lower than those in other areas. During 2002, the hospital admission rate for the western region of the United States (955.4 per 10,000 population), was 18.6 percent below the national average (1,174.6 per 10,000). During the same period, the mean hospital stay in the west (4.4 days) was 8.9 percent below the national average (4.9 days) [17].

Reductions in health care utilization brought about by managed care in the western United States have been adopted in other areas of the nation and the world. Physician profiling and the development of preferred provider arrangements with long term care providers have been employed by providers and payors to constrain hospital utilization and related to costs [18].

## The Decline of Managed Care

In retrospect, it appears that the success of managed care in the United States during the last decades of the twentieth century also led to its undoing. Health care is a dynamic sector of many national economies. Upon review of the American experience, it seems that a number of factors contributed to the decline of this approach.

The initial wave of opposition to managed care appeared as challenges to control of health care utilization, such as choice of providers. Physicians were never reconciled to allowing insurance plans to choose practitioners and hospitals. During the 1990's, a wave of class action litigation was directed at the ability of plans to direct referrals for health services. The plaintiffs in these cases, including consumers, physicians, and other providers, sought financial damages and injunctions against managed care business practices. Although courts in the United States were reluctant to declare the costs control methods of plans illegal, the full range of litigation supported negative perceptions of managed care among consumers and businesses [19].

During the late 1990s, the legal reaction against managed care in the United States was accompanied by deteriorating relationships between plans and health care providers. Increased consumer dissatisfaction with the business practices of plans, including apparent arbitrary denials of service and failure to pay claims promptly, added fuel to provider complaints about low payment rates. Providers also objected to the terms of risk contracting agreements which required them to carry a significant burden of pharmaceutical costs and other expenses. All of these developments generated a wave of opposition to managed care by hospitals and physician groups. These activities frequently resulted in the termination of contracts and network instability [20,21].

During the second half of the 1990's, consumer litigation and provider reactions against managed care caused these many of these plans to change their traditional business approaches. In order to regain the favor of consumers and providers, plans in many communities loosened controls on provider utilization. Plans relaxed the use of physicians as gate keepers and allowed consumers direct access to specialists. Requirements for referrals to other types of services were loosened. Stringent authorization procedures for use of hospital emergency departments and for certain surgical procedures were also relaxed. This process amounted to a substantial movement away from the aggressive health care management approaches which had supported the rise of this approach [22].

To replace these utilization control mechanisms, many managed care plans introduced mechanisms that included more interaction with subscribers, such as case management and electronic information exchange. Subscribers were encouraged to modify health related behaviors by visiting web sites and obtaining additional information concerning preventive care. In many communities, managed care plans placed increased emphasis on wellness programs and disease management. The industry was shifting its focus from controlling utilization to influencing it.

The retreat from direct management of health care utilization toward softer approaches led to a general weakening of accountability to consumers. With fewer direct controls in place and greater reliance on indirect mechanisms the assessment of performance of individual plans by businesses and government became more difficult [8].

Related to these approaches, some managed care plans adopted business strategies which placed greater emphasis on profitability. Chief among these was a movement away from contracting for high risk populations. Facing increased dissatisfaction from consumers and greater competition from traditional insurance in the market place, private plans in many areas increasingly focused on lower risk populations as a means of improving profitability. As a result, many private managed care plans withdrew from participation in public managed care programs with high risk populations such as the elderly (Medicare) and the indigent (Medicaid). Between 1998 and 2000, the number of plans serving Medicare patients declined by 20 percent. By 2002, total Medicare managed care enrollment was lower than it had been in 1997. Between 1998 and 2000, the number of insurance plans participating in Medicaid declined by 15 percent. The development had a major impact on State governments because more than half of all Medicaid recipients were enrolled in managed care [23].

Increased efforts by managed care plans to compete in the United States health care marketplace also led to the adoption of other business strategies characteristic of traditional insurance. These included reduced a greater emphasis of raising premiums to support profitability. This approach effectively passed along a higher proportion of health care expenses to subscribers. Managed care plans also catered more to consumer preferences in order to expand market share [24].

All of these activities effectively changed managed care from its traditional structure in the United States. As the focus on continuity of care and regulation of utilization diminished, managed care plans became more like other types of insurance such as indemnity plans and preferred provider organizations. As choice of providers was substituted for gate keeping, physicians and increased premiums took the place of utilization controls, the line between managed non managed care became blurred.

**Figure 1:**
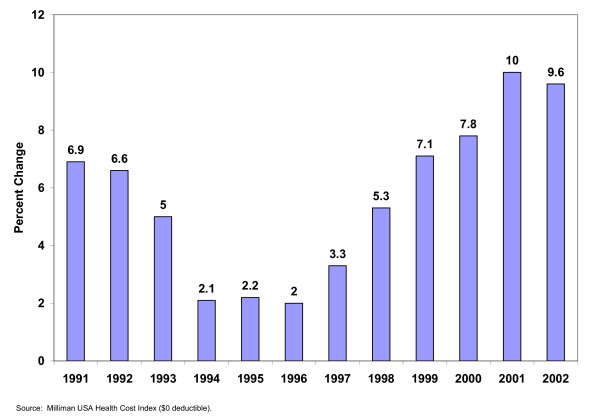
Annual percent change per capita health care spending United States 1991 – 2002.

This entire process amounted to a decline of managed care shortly after the approach had reached its highest level of acceptance. This decline was reflected in a decreased ability of managed care to restrain costs, a major reason for the increased use of this form by payor during the 1980s and 90s. As a result of this development, a major barrier to health care spending was removed and per capita expenditures began to rise. This situation is illustrated by changes in annual per capita health care spending for all payors in the United States, summarized in Figure 1.

This information identifies both the zenith and the decline of managed care. Between 1994 and 1996, the impact of this approach reached a high point, as annual increases in per capita spending declined to only about two percent per year. The decline of managed care during the late 1990s produced a rapid erosion of this position. Annual increases in per capita spending nearly quadrupled to almost eight percent by 2000 and kept increasing after the turn of the century.

**Figure 2:**
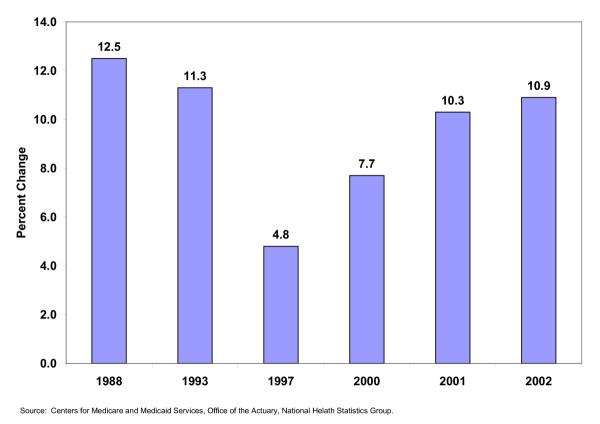
Annual percent change per capita spending for private health insurance United States 1988 – 2002.

These developments were paralleled by in annual increases of changes in per capita spending for private health insurance, the area of the economy in which managed care developed prior to the 1970s. After declining to less than five percent in 1997, annual increases in per capita expenses for private health insurance rapidly recovered to over 10 percent after the turn of the century. These developments are summarized in Figure 2.

The increases in pre capita health expenditures identified in these tables occurred because of a retreat from traditional cost controls by managed care plans and because of the transfer of many expenses to consumers. As previously noted, changes in the behavior of these plans in some metropolitan areas included less reluctance to raise premiums, rather than contain costs, in order to avoid operating losses. Many of the 'soft' programs that were added in order to compete with traditional health insurance were offered at an additional cost.

This information concerning the impact of managed care on health care expenses does not identify the impact of the decline of the approach on continuity of care and other outcomes, for which quantitative data are not available. It might be assumed that, with increased emphasis on consumer choice and less attention to control of referrals and provider participation, the integrity of care across health systems also deteriorated during this period.

All of these developments effectively moved managed care plans in the United States closer to traditional insurance plans in their behavior and impact on health care expenditures. They undermined the ability of this approach to differentiate itself from traditional health insurance as a mechanism which actively managed care and contained costs. They made it considerably more difficult for the approach to exert a direct impact on health care outcomes. Ironically, these characteristics had been the major selling points of the approach since its inception.

It should be noted that, during this period, traditional insurance plans have also moved toward managed care by adopting features of health maintenance organizations such as utilization controls. As a result, the border between these two types of insurance is now almost non existent.

The change in the character of managed care also had profound implications for the future of health care cost containment in the United States. It deprived government and the private sector of one of their most powerful weapons in restraining expenditures. It also signaled an important change in the direction off public and private sector health policy. The movement of managed care away from utilization controls and toward higher premiums shifted the burden of health policy toward the consumer. It suggested that payors would support consumers in shaping health care and the organizations that provide it, rather than having the payors assume leadership. This change would lead to important developments in the policy environment of this sector.

## The Rise of Consumer Driven Health Care

The decline of managed care as the major driver of health care policy and reimbursement within the United States has opened the way for new forces to shape this area. The nature of these forces became visible in the late 1990s as managed care plans shifted responsibility for health care decision making to consumers. The resulting annual increases in health care expenditures, summarized in Figure 1 and 2, were also effectively shifted to consumers through higher premiums, deductibles, and copayments. For example, in preferred provider organizations, the most widely used health plans, single coverage deductibles increased more than 50 percent between 2002 and 2003. More employers began offering high deductible plans. The rate of increase of out of pocket spending has increased every year between 2000 and 2003 [25,26].

The rise of increased consumer involvement in health care in the United States has developed on several fronts. At the broadest level, it has taken the form of a greater consumer role in decision making concerning treatment. In the late 1990's the use of television, newspapers, and electronic media to market health care to consumers became pervasive. Drug companies became major users of this approach. They have conducted enormous media campaigns to promote the use of sexual stimulants, allergy medicines, and cosmetic treatments. Access to large marketing budgets has made it possible for these companies to reach millions of consumers with their messages. Local and regional diagnostic firms have also marketed magnetic resonance imaging and whole body scanning through similar campaigns [27].

These efforts have been successful because they gone directly to users of health care. They have circumvented insurance companies, managed care, and even physicians. Listeners are urged to 'ask' or 'tell' their doctor to prescribe any number of medications or tests. The clear message of all of these initiatives is for individual consumers to take a greater role in health care decision making. Greater user involvement in health care that was stimulated by the decline of managed care, as well as media initiatives of the pharmaceutical industry and other groups, have led directly to the development of a new type of health insurance in the United States, the consumer driven health plan. These mechanisms complete the change initiated by the decline of managed care by directly assigning health care decision-making to consumers.

Consumer driven health plans are designed to address the major objectives of managed care, development of healthful behaviors and containment of health care costs. Components of consumer driven plans usually include the following.

- High deductibles as incentives for greater consumer participation in the cost of care

- Catastrophic coverage for high cost services such as inpatient hospitalization

- Consumer savings accounts for funding of prevention and screening services

- Procedures for roll over of unused savings account balances to future time periods

- Support for consumer decision making through availability of internet based information concerning health care risk factors and provider outcomes

- Tracking of employee health expenses through the system [28,29].

These components have been designed to replace structures of managed care plans which addressed the same objectives. Encouragement of healthful behaviors and health status objectives are addressed through consumer savings accounts, rollover provisions, and internet based information. These provisions have effectively transferred responsibility for the management of care from the primary care physician gatekeepers employed by managed care plans to the consumer. This transfer has been supported by a combination of financial incentives and electronic data [28,30].

Consumer driven health plans also contain provisions for cost containment. High deductibles and catastrophic coverage are intended to limit expenditures by payors. These provisions effectively reduce expenses for many of the pharmaceutical and ambulatory care expenses currently being pushed by media advertising. By excluding payor reimbursement for them, these plans are transferring these expenses to the consumers, or providing incentives to eliminate the purchases altogether. This amounts to a cost containment approach very different from the utilization controls employed by managed care plans [31].

The implementation of consumer driven health plans has generated extensive controversy in the American health care system. Supporters of this approach have argued that it is logical because it places major responsibility for health care decision making in the hands of the party who will be most influenced by those decisions, the consumer. They have emphasized that, under these plans, positive health care behaviors and decision making are rewarded by fewer out of pocket expenses. They have also noted that consumers are provided with extensive electronic information needed to support effective decision-making [30,32].

Supporters of consumer driven plans also have suggested that these approaches include realistic mechanisms for health care cost containment. They have pointed out that managed care relied on limitations on the utilization of care to limit spending, while consumer driven plans assign these decisions to users of care. They have suggested that the new approach can satisfy both the payor and the consumer, by limiting insurance expenses for care and allowing individuals to purchase additional services [28].

Opponents of consumer driven health care have argued that this approach is inferior to managed care, that it is a mechanism for employers and payors to abdicate their responsibilities. They have suggested that, rather than supporting consumers with the advice of physicians and other health care providers, it turns them loose to make decisions on their own. They have indicated that a set of financial incentives and internet based information is no substitute for the relationship between a patient and a caregiver [29].

Opponents of consumer driven plans also argued that the real purpose of these approaches is financial, that they are better identified as defined contribution plans. Opponents of these plans suggested that they are really covers for the abdication of financial responsibility by payors. They have argued that the major purpose of these plans is to shift health care costs from employers to employees. They have contended that high deductibles and catastrophic coverage are the real solution for employers and payors seeking to reduce premiums, regardless of the impact on subscribers [30,31].

The debate concerning consumer driven health care is probably only beginning in the United States. It bears similarities to the discussion surrounding the rise of managed care in the 1970s and 80s. One important difference between these situations may be the economic background. The ascendancy of managed care developed against the background of an upturn. Consumer driven care is developing at a time of economic instability and limited resources. Indeed, these conditions may be supporting the rise of the new approach.

A review of the current status on consumer driven plans suggests that they are still evolving. Many of these plans are still developing their own provider networks. Others are partnering with existing plans in order to expedite the process. Some plans are developing generic fee schedules for services and allowing consumers to develop their own networks. Consistent with this approach, consumers cover additional health care costs from their own resources [31].

The development of information infrastructure has become an important part of the implementation of consumer driven plans. In order to involve consumers in a meaningful way, plans must make available extensive electronic data to support decision making. These data include a variety of online sources of information including many types of research. They also must include financial information concerning provider prices and discounts. In order to enable consumers to participate in health care decision making on a continuing basis, plans must also make available data concerning consumer accounts which identify the impact of choices on available funds. The plans which are entering the consumer driven market in a serious manner must have all of these information resources available. They require an extensive investment in data infrastructure [31].

**Figure 3:**
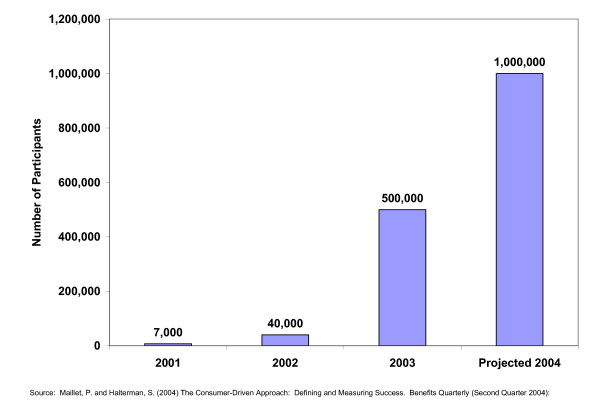
Participants in consumer drive health plans United States 2001 – 2004.

The implementation of consumer driven health plans is proceeding rapidly in the United States. Because of the size of the American population, it still occupies a relatively small proportion of the health care market. The data in Figure 3 demonstrate that, by 2004 enrollment in consumer driven health plans is projected to reach 1,000,000. This enrollment is still dwarfed by existing traditional insurance and managed care populations.

**Figure 4:**
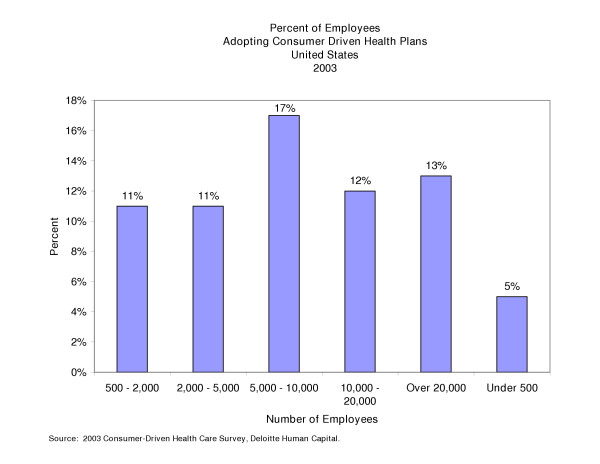
Percent of employees adopting consumer driven health plans United States 2003.

The distribution of consumer driven health care enrollment across employers is summarized in Figure 4. This data indicate that market penetration by these plans is still modest, but that it has established a foothold in a wide range of employers [28]. This suggests that interest in these plans is not limited by employer size.

It should be noted that these data reflect the growth of consumer driven plans among private insurance plans. This approach has not yet been tested with Medicare or Medicaid populations. These effort may require the development of new designs for plan components, especially those which involve consumer decision making, because of differences in health care mind sets and behaviors among the elderly and the indigent.

The future of consumer driven health care in the United States is difficult to predict. Additional time and utilization data will be required to determine whether this approach generates sufficient enrollment to develop into a major force within the health care system of the United States and whether other nations adopt it.

## Implications for Europe

The recent development of managed care and consumer driven health care in the United States has important implications. Rightly or wrongly, the American health care system has historically been a source of approaches for implementation in Europe and elsewhere. The amount of change and innovation that has marked this system in recent years has generated more than enough material for consideration by the international community.

One of the most significant developments in the United States health care system in the past several years has been the decline of managed care. This development probably came as a surprise to health care policy makers elsewhere in the world. In its early days, managed care had great promise for addressing the two most important objectives of health care planning, improving patient outcomes while containing costs. During the 1980s, this approach seemed to be on its way toward meeting this objective in the American health care system. Managed care penetration of the private insurance, Medicare, and Medicaid markets were increasing substantially and previous annual increases in per capita health care expenditures were declining.

The reasons for the decline of managed care probably emanate from conflicting forces within the American health care system and the environment surrounding it. These include the desire to support buyer preferences and the need to restrain increases in health care expenditures at the same time. In the American system during the late 1990s, cost containment lost out. The lesson that may emerge from this is one of realism. You cannot have your cake and eat it too.

At the same time, this story is more complicated than a struggle between choice and cost containment. It is part of a rising interest in consumer choice within the wider health care environment and the economy of the United States. Pharmaceutical companies and other providers of health services have recognized this and begun direct marketing to consumers through the media. This development has involved bypassing physicians and going directly to users of care, just as consumer driven care involves bypassing managed care and its utilization controls. These developments suggest that consumer driven health care in the United States is not an isolated event, but part of a wider trend.

These developments pose a major dilemma for health policy makers. The parameters of this dilemma may differ among nations. It can be suggested that, in the United States, advocates of managed care should not have 'bailed out' so quickly when consumer dissatisfaction began to increase. This assumes that purchasers of care will ultimately side with cost containment.

The preceding information demonstrates that the following chain of events has occurred in the United States. Each of these events was related to wider developments in the American economy.

- Managed care helps reduce health care cost increases

- Increased managed care results generates popular dissatisfaction

- Managed care plans reduce controls and increase prices

- Health care costs increase substantially

- Consumer driven plans provide a mechanism for government and employers to unload health care costs on consumers

This important juncture is where Europe and the rest of the world may have an advantage over the United States. It is clear that Europe has the opportunity to invest in managed care and to use this approach to restrain costs and improve patient outcomes. In so doing, European nations would stay with the first step on the preceding chain of events and avoid the disadvantages of the remaining ones.

But before we elaborate further on possible implications for Europe, we should emphasize that the stage for the health care actors in Europe is quite different from the one in the USA. Health care systems in Europe vary greatly among countries, but in general European health care systems have some characteristics that make them different from the system(s) across the Atlantic. First, Europe has a highly valued tradition of universal health care coverage and governmental administration. Furthermore, Europeans tend to entrust responsibility to the (central) government and not to private agencies. Finally, competition among providers and purchasers is restricted by highly structured conditions by the government.

The above comes down to the point that in health care Europeans have difficulties getting market elements off the ground. They do not seem to trust the invisible hand (of the market). This has probably resulted in a slower rate of system innovation, but fewer policy development and implementation errors, in these countries [33,34].

Germany, Switzerland and The Netherlands are nowadays starting to adopt managed care (tools) with caution, focusing on support for patient outcomes and cost containment. At the same time, Sweden and the United Kingdom, implemented dramatic changes very rapidly. Obviously, Europe also offers the opportunity for a number of different approaches to this issue to develop simultaneously [35,36].

Any evaluation of the potential impact of consumer driven health care in Europe must address specific aspects of this approach. For example, what would be the implications of providing information concerning pharmaceuticals directly to consumers in Europe? It could also be conjectured that Europeans might prove to be more deliberate and conservative consumers of pharmaceuticals than Americans. It could be suggested that Europeans would make more extensive use of information before reacting to the first television commercial and asking their physicians for Viagra or Allegra, or some other drug. At the same time, however, the answers to these questions are not clear. They may vary substantially among European countries [36,37].

A more important question regarding consumer driven health care concerns the implications of exchanging free choice of providers for higher cost sharing by consumers. In societies with established medical networks which have become acclimated to managed care, the free choice option might have little impact. At the same time, the recent strength of the European economies and the euro might reduce the impact of higher cost sharing and make free choice of providers more affordable [38,39].

Another area of consumer driven health care that deserves evaluation is the potential impact of providing health information directly to consumers on the use of services and costs. In the United States, the effectiveness of this approach depends greatly on the extent to which consumers read, evaluate, and act on health information provided through websites and other electronic media. It is clear that Europeans probably make as much use of the internet as Americans, however, it remains to be seen what the impact of electronic medical information will be on consumer behaviors in these countries [40].

The answers to these specific questions could vary substantially across the European continent. The degree to which managed care is retained could be related to the commitment of individual governments, the influence of medical establishments, and the availability of disposable income. From a policy making standpoint, this issue probably requires evaluation on a nation by nation basis.

From an international standpoint, the potential impact of the decline of managed care and the rise of consumer driven health care in the United States is a fascinating issue. It is still too early to determine what the impact of these developments will be on the rest of the world. One answer to this question is probably that changes in health care policy require adequate information on the effectiveness of the alternative being considered, as well as the status quo. It appears that the difficulties with managed care experienced by the United States during the 1990s have prompted a rapid movement away from this form, without a clear idea of the effectiveness of consumer driven care, or other alternatives. Health care has too much of an impact on large populations and on national spending to approach it by 'looking before leaping'.

All of this suggests that Europe and the rest of the international community are in an excellent position to profit from the American experience without much risk. They can view what is going on with critical eyes and reach their own conclusions about whether the transition from managed to consumer driven care supports patient outcomes or not. They can monitor the data and determine which of these forms has greater potential for cost containment. The opportunities for learning may increase as financial pressures place greater pressures on the American system for cost containment.

## References

[B1] Reinhardt UE, Hussey PS, Anderson GF (2002). Cross – National Comparisons of Health Systems Using OECD Data, 1999. Health Affairs.

[B2] Anderson GF, Reinhardt UE, Hussey PS, Petrosyan V (2003). It's the prices, stupid: Why the United States is so different from other countries. Health Affairs.

[B3] Kelly RR (2004). America's health care mess (dirty little secrets few talk about). Hawaii Medical Journal.

[B4] Levit K, Smith C, Cowan C, Sensenig A, Catlin A (2004). Health spending rebound continues in 2002. Health Affairs.

[B5] Hoadley JF, Cunningham P, McHugh M (2004). Popular Medicaid programs do battle with state budget pressures: perspectives from twelve states. Health Affairs.

[B6] Fong T (2004). Medicaid under the knife?: Congress considering funding cuts in program?. Modern Healthcare.

[B7] Marquis MS, Long SH (1999). Trends in managed care and managed competition, 1993–1997. Health Affairs.

[B8] Draper DA, Hurley RE, Lesser CS, Strunk BC (2002). The changing face of managed care. Health Affairs.

[B9] Jensen G, Morrissey MA, Gaffrey S, Liston DK (1997). The new dominance of managed care: insurance trends in the 1990's. Health Affairs.

[B10] Zarabozo C (2000). Milestones in Medicare managed care. Health Care Financing Review.

[B11] Cagey C (2000). Health reform, year seven: observations about Medicaid managed care. Health Care Financing Review.

[B12] Jensen G (1997). The new dominance of managed care: Insurance trends in the 1990s. Health Affairs.

[B13] Gabel JK, Hurst K (1998). Health benefits in 1998: Executive summary.

[B14] Gold M (2001). Medicare + Choice: An interim report card. Health Affairs.

[B15] Holahan J, Suzuki S (2003). Medicaid managed care payments and capitation rates in 2001. Health Affairs.

[B16] (2002). Medicare and Medicaid Statistical Supplement. Health Care Financing Review.

[B17] DeFrances CJ, Hall MJ (2004). 2002 National hospital discharge survey. Advance Data.

[B18] Lagoe RJ, Westert GP (2004). Improving outcomes with community wide distribution of health care data. Health Care Management Review.

[B19] Havighurst CC (2001). Consumers versus managed care: The new class actions. Health Affairs.

[B20] Strunk B, Devers K, Hurley R (2001). Health plan – provider showdowns on the rise. Washington, DC: Center for Studying Health Systems Change.

[B21] Kelly PM (2004). Will employee choice and defined contribution health plans salvage the embattled managed care system?. Benefits Quarterly.

[B22] Bachman RE (2004). Consumer-driven health care: The future is now. Benefits Quarterly.

[B23] Interstudy (2001). Competitive Edge 11.1, Part II: HMO Industry Report.

[B24] Lair T (2004). Redefined contribution health care. Benefits Quarterly.

[B25] Smith C, Cowan C, Sensenig A, Catlin A, the Health Accounts Team (2005). Health spending growth slows in 2003. Health Affairs.

[B26] Henry J (2003). Kaiser Family Foundation and Health Research and Educational Trust. Employee Health Benefits: 2003 Annual Survey.

[B27] Marquis MS, Long SH (1999). Trends in managed care and managed competition, 1993–1997. Health Affairs.

[B28] Maillet P, Halterman S (2004). The consumer-driven approach: Defining and measuring success. Benefits Quarterly.

[B29] Meyer J (2004). Consumer-driven health plans: Design features to promote quality improvement. Benefits Quarterly.

[B30] Beauregard TR (2004). Consumer-driven health care: Tangible employer actions. Benefits Quarterly.

[B31] Christianson J, Parente ST, Taylor R (2002). Defined-contribution health insurance products: Development and prospects. Health Affairs.

[B32] Marhula D, Shannon E (2000). Defined contribution defined: Health insurance for the next century.

[B33] Rienhardt UE, Hussey PS, Anderson GF (2004). US health care spending in an international context. Health Affairs.

[B34] van de Ven WPMM, van Vliet RCJA, Lamers LM (2004). Health adjusted premium subsidies in the Netherlands. Health Affairs.

[B35] Stevens S (2004). Reform strategies for the English NHS. Health Affairs.

[B36] Christian-Herman J, Emons M, George D (2004). Effects of generic – only drug coverage in a Medicare HMO. Health Affairs.

[B37] Wallack SS, Weinberg DB, Thomas CP (2004). Health plans' strategies to control prescription drug spending. Health Affairs.

[B38] J.P. Newhouse and the Insurance Experiment Group (1993). Free for all? Lessons from the RAND Health Insurance Experiment.

[B39] J.P. Newhouse (1981). Some interim results from a controlled trial of cost sharing in health insurance. New England Journal of Medicine.

[B40] Riggs DL, Holdsworth SM, McAvoy DR (2004). Direct-to-consumer advertising: Developing evidence-based policy to improve retention and competition. Health Affairs 2004.

